# Author’s Reply to “Concerns regarding Validity of the Use of Bean Extract-Based Gargle for COVID-19 Diagnosis”

**DOI:** 10.1128/spectrum.01070-22

**Published:** 2022-07-05

**Authors:** Joseph Kwon, Euna Ko, Se-Young Cho, Young-Ho Lee, Sangmi Jun, Kyuhong Lee, Eunha Hwang, Bipin Vaidya, Jeong-Hwan Hwang, Joo-Hee Hwang, Namsu Kim, Mi-Kyung Song, Hye-Yeon Kim, Dai Ito, Yuxi Lin, Eunae Jo, Kyeong Eun Yang, Hee-Chung Chung, Soyoung Cha, Dong Im Kim, Yoon-Sun Yi, Sung-Ho Yun, Sun Cheol Park, Sangmin Lee, Jong-Soon Choi, Dal Sik Kim, Duwoon Kim

**Affiliations:** a Korea Basic Science Institute, Daejeon, Republic of Korea; b Chonnam National University, Gwangju, Republic of Korea; c Jeonbuk National University Medical School and Hospital, Jeonju, Republic of Korea; d Korea Institute of Toxicology, Daejeon, Republic of Korea; e Daegu Gyeongbuk Institute of Science and Technology, Daegu, Republic of Korea; f BioApplications Inc., Pohang, Republic of Korea; g BIO3S, Inc., Gwangju, Republic of Korea; University of Georgia

**Keywords:** COVID-19, SARS-CoV-2, oral virus, rapid diagnostic test, sensitivity, specificity

## REPLY

We appreciate Hong et al. for their precious time in reviewing and commenting on our paper entitled “Bean extract-based gargle for efficient diagnosis of active COVID-19 infection using rapid antigen tests” (1) and would like to thank the editor for the opportunity to respond to the comments. The comments led us to reduce misunderstandings among readers regarding the validity of the test method and interpretation of the results. The point-by-point responses are provided below.

First, as stated by the commenter, the coronavirus disease 2019 (COVID-19) testing PCR protocols of Allplex 2019-nCoV real-time PCR and a STANDARD M nCoV Real-Time Detection kit as applied for nasopharyngeal swab (NPS) and Beanguard gargle (BG) specimens, respectively, can attribute to the difference in Ct values of NPS-RT-PCR and BG-RT-PCR. However, our study intended to improve the performance of Ag-RDTs using a bean extract-based gargle (BG). Ag-RDTs provide either positive or negative test results, not compare Ct values. In Fig. 2A, Fig. S1A, Fig. S2A to D, and Fig. S3, Ct values were displayed only to demonstrate the distribution of Ct values. Moreover, different PCR protocols were applied separately for NPS and saliva by Green Cross Laboratories, South Korea. According to the manufacturer’s instructions (2–3), the Ct cutoff values of ≤40 and ≤36 were considered positive for Allplex 2019-nCoV real-time PCR and STANDARD M nCoV Real-Time Detection kit, respectively. Our study mainly focused on comparing the effectiveness of BG-RT-PCR with NPS-RT-PCR in terms of sensitivity and specificity (considering the test results as positive or negative), not to compare Ct values between NPS-RT-PCR and BG-RT-PCR.

Second, regarding the reference standard adopted for the study, we already explained in our current study (1) that if bean gargle and saliva samples were simultaneously collected from the same patient at the same sampling time, the sequential order of two sample collection methods affected the amount of collected virus and virus diagnostic test results. Even though the commenter’s opinion is correct in the sense that the comparison between BG and gargled saliva was beneficial, it was not practically feasible to collect and compare BG and saliva samples from the same patient. Therefore, NPS-RT-PCR, the FDA-recommended reference standard method, was adopted to compare the performance of BG-Ag-RDT and BG-RT-PCR and presented the result in Table 2 in our research paper.

Third, like all other studies, our study also has certain limitations that we mentioned in the manuscript. The number of different variants, such as Alpha and Delta, and asymptomatic cases as well were not sufficiently available to generalize our test result. Due to the nonpurposive sampling method, the required number of asymptomatic cases was not available for study to represent the whole population. Moreover, the handling and conducting of a clinical trial of the SARS-CoV-2 detection method itself were challenging when the trial was being conducted. We will conduct a further clinical trial in accordance with the guidelines of the Ministry of Food and Drug Safety, South Korea.
